# Early Intervention in Class II Division 1 Malocclusion Using an Activator Appliance: A Case Study

**DOI:** 10.7759/cureus.67244

**Published:** 2024-08-19

**Authors:** Mrudula Shinde, Pallavi Daigavane, Ranjit Kamble, Nikhil Kumar, Nishu Agarwal

**Affiliations:** 1 Orthodontics and Dentofacial Orthopaedics, Sharad Pawar Dental College and Hospital, Datta Meghe Institute of Medical Sciences, Wardha, IND; 2 Orthodontics and Dentofacial Orthopaedics, Kusum Devi Sunderlal Dugar Jain Dental College & Hospital, Kolkata, IND

**Keywords:** mandibular rotation, codylar growth, activator, class ii div 1 malocclusion, phase 1 treatment, myofunctional appliance

## Abstract

The field of orthodontics has traditionally been regarded as the primary specialty within dentistry. As per Dr. Tweed's recommendation, historical treatment approaches often entailed the extraction of four premolar teeth. Nonetheless, early orthodontic intervention can frequently obviate the necessity for extractions, thus preserving the integrity, functionality, and aesthetic appeal of the dentition. This case report details a non-extraction approach for managing a developing skeletal Class II malocclusion, characterized by a skeletal disharmony between the maxilla and mandible. The chosen treatment option is influenced by factors such as the patient's age, growth potential, the severity of the malocclusion, and the patient's adherence to the prescribed treatment regimen. Myofunctional appliances have been identified as effective in addressing Class II Division 1 malocclusion resulting from mandibular retrusion.

## Introduction

Orthodontics aims to address patients' concerns, achieve optimal functional outcomes, and improve aesthetic results. There has been significant debate among orthodontists regarding the ideal timing for initiating treatment for patients with malocclusions. It is crucial to manage the development of dentition and occlusion during the growth phases of children and adolescents. Early intervention plays a vital role in interceptive orthodontics, aiming to correct skeletal and dental malocclusions [1].

Early detection of malocclusions brings significant benefits. In younger patients, interceptive orthodontics is best approached with simplicity [2]. Class II Division 1 malocclusion ranks among the most prevalent types of malocclusion worldwide. The prevalence of Class II malocclusion in India varies depending on the region. Generally, Class II malocclusion affects a significant portion of the population, with estimates ranging from 10% to 30%. McNamara identifies mandibular retrusion as a common feature in growing children with this malocclusion [2]. It affects 15% of the global population and often involves a skeletal mismatch between the upper and lower jaws, potentially due to a protrusive upper jaw, a recessed lower jaw, or both.

Growth modification is an interceptive treatment for developing Class II skeletal conditions, aiming to improve the skeletal relationship by influencing facial growth and altering the jaws' size, orientation, and position [3,4]. Orofacial myofunctional therapy, increasingly integrated into orthodontic practice, addresses facial muscle imbalances, educates on correct tongue positioning, and enhances coordination among the lip, cheek muscles, and tongue, influencing dental growth [5,6]. Prefabricated functional appliances have shown favorable outcomes in children with Class II Division 1 malocclusions. This article discusses treatment strategies for Class II Division 1 malocclusion in young patients.

## Case presentation

An 11-year-old male patient presented to the Department of Orthodontics and Dentofacial Orthopedics at Sharad Pawar Dental College and Hospital with a primary complaint of upper front teeth protrusion. A detailed habit history and clinical assessment were conducted, along with the collection of pre-treatment records, including radiographs, study models, and photographs.

**Figure 1:**
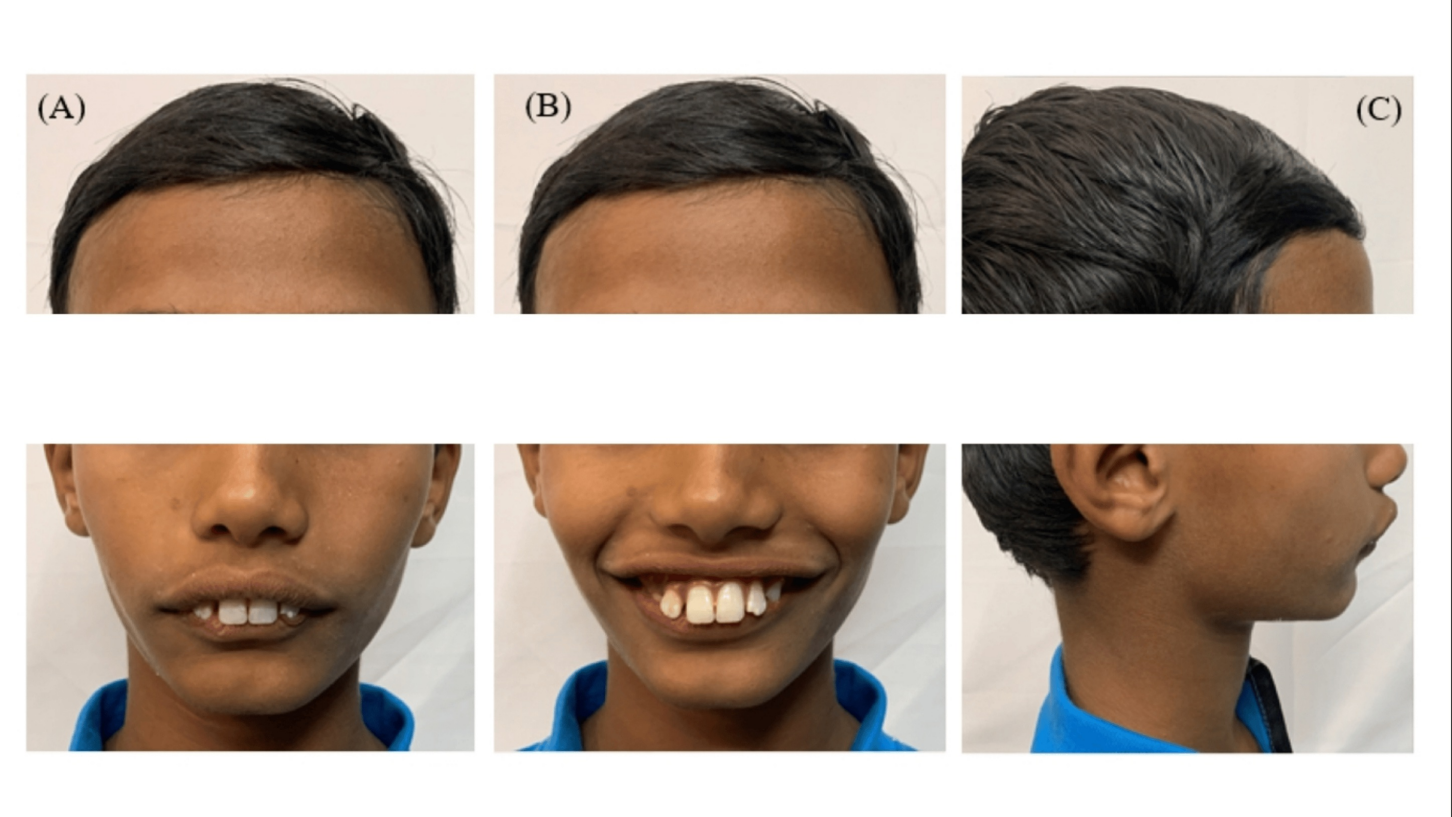
Pretreatment extraoral photographs showing the (A) frontal, (B) smiling, and (C) left lateral profiles

The clinical examination revealed a symmetrical mesoprosopic face form and incompetent lips. A profile examination showed a convex form with posterior divergence due to a retrognathic mandible, deep labiomental fold, increased lower anterior facial height, hyperactivity of the mentalis and buccinator muscles, and hypotonic upper lip. The lips were incompetent and protrusive, with a 12 mm interlabial gap when smiling, and the lower lip was everted (Figure 1).

**Figure 2:**
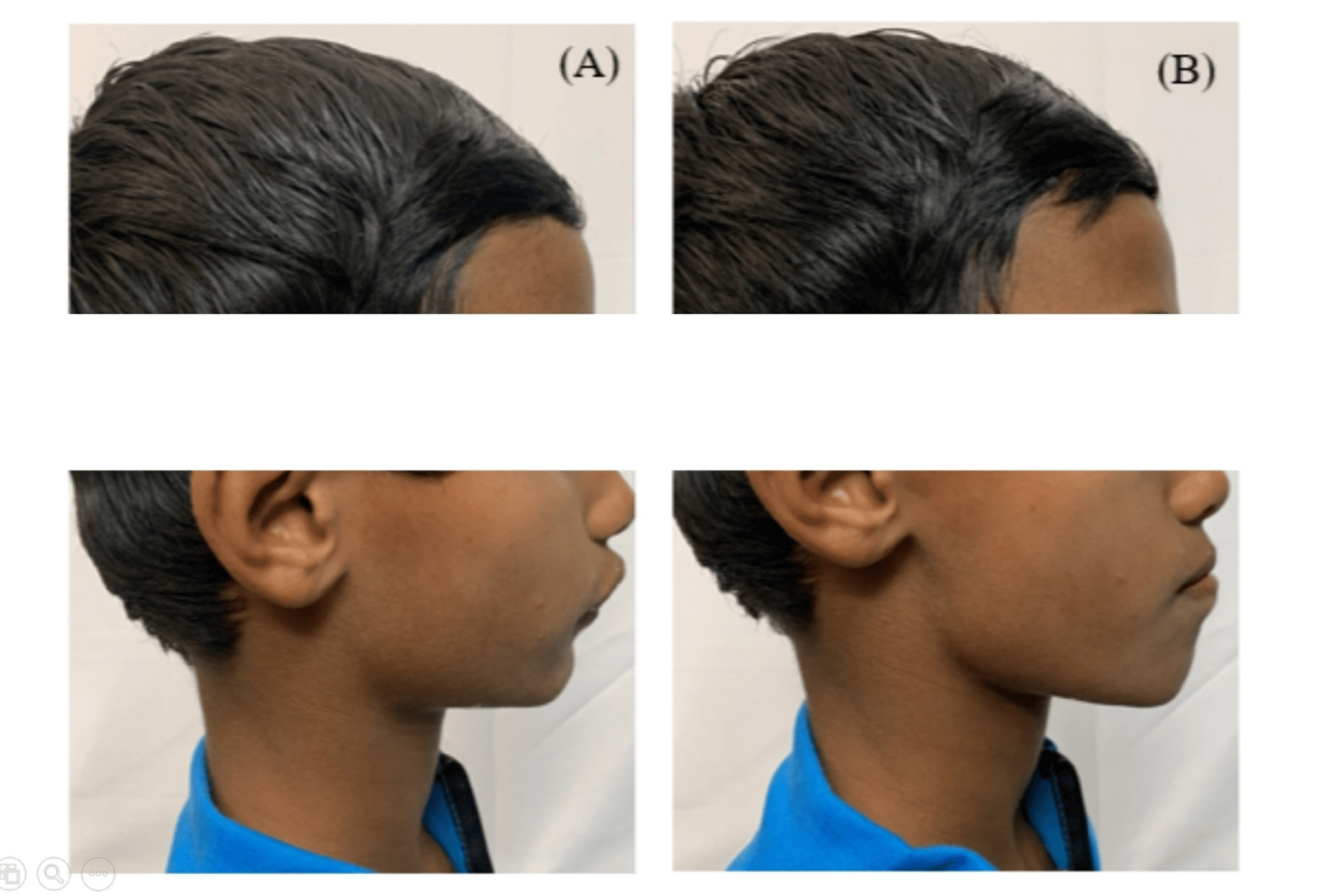
Positive visual treatment objective showing the (A) before and (B) after treatment profiles

The evaluation of the temporomandibular joint revealed normal movements. The visual treatment objective indicated a significant profile improvement, ensuring a successful outcome (Figure 2).

**Figure 3:**
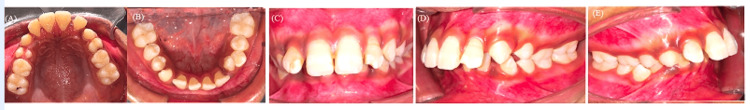
Pretreatment intraoral photograph of the (A) maxillary arch, (B) mandibular arch, (C) anteriors in occlusion, (D) left molars in occlusion, and (E) right molars in occlusion

Upon intraoral examination, it was observed that the patient was in the late mixed dentition stage, with emerging maxillary canines and premolars and retained second deciduous molars present on both sides of the maxillary arch. The maxillary dental arches displayed a "U" shape, with proclined anterior teeth and spacing noted. Closure of the mandibular incisors resulted in impingement on the palatal tissue. The patient exhibited a Class II Division 1 occlusion with an overbite of 8 mm and an overjet of 11 mm. Molar and canine relationships were observed to be end-on, indicating a tendency towards Angle's Class II relation on both sides (Figure 3).

**Figure 4:**
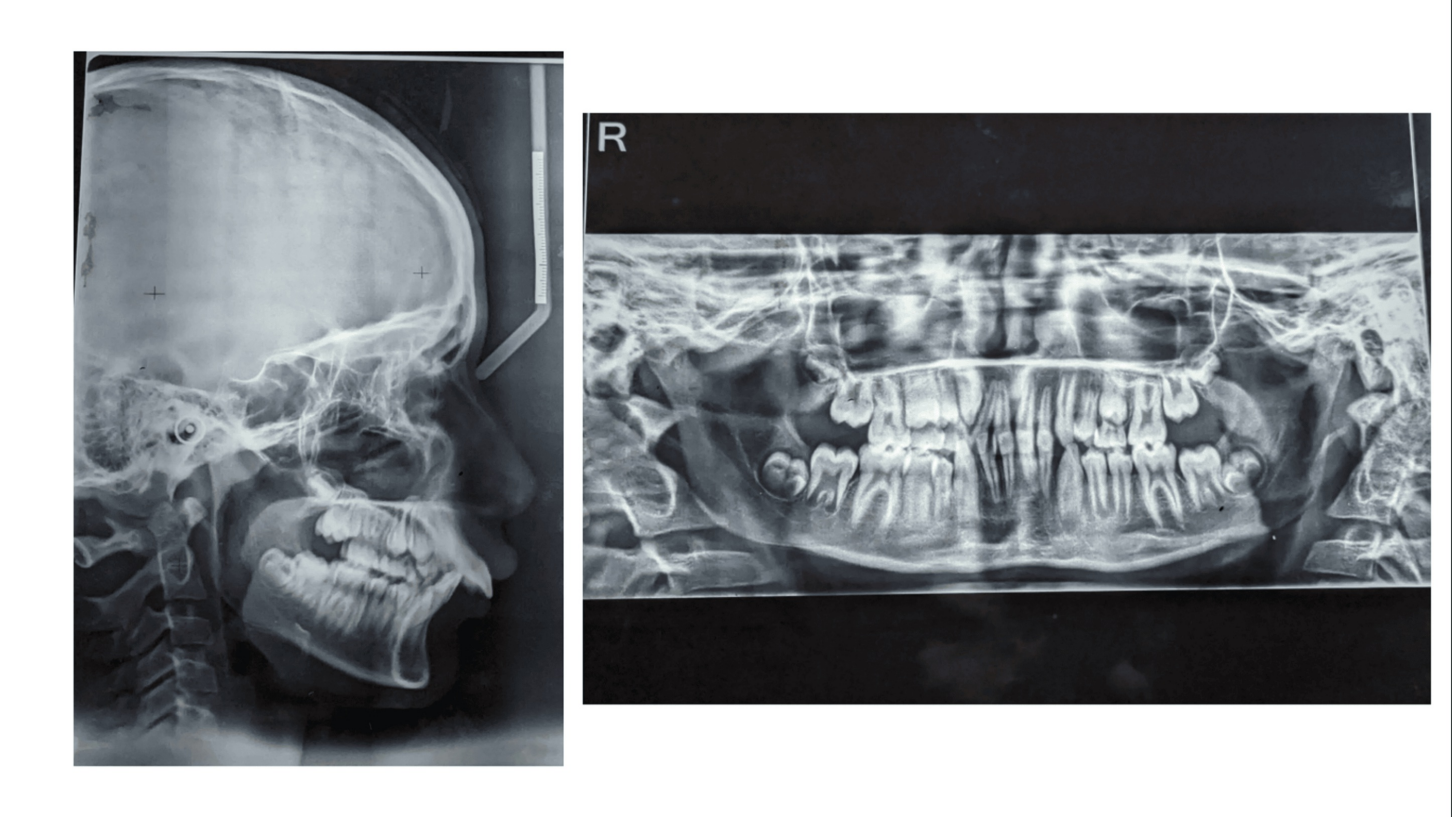
Pretreatment lateral cephalogram and orthopantomogram

During the prepubertal growth phase, the patient was identified at Cervico Vertebrae Maturation Index stage 2. This stage is marked by the flat morphology of the inferior margins of cervical vertebrae C2, C3, and C4. The bodies of C3 and C4 vertebrae are trapezoidal in shape, narrowing progressively from posterior to anterior along their superior borders. These findings were derived from a complementary cephalometric assessment (Figure 4).

The orthopantomogram findings indicate that the patient's dental anatomy demonstrates continuous posterior and lower boundaries, appropriately positioned condyles, sufficient alveolar bone height, and commendable root alignment. The assessment confirms the presence of skeletal Class II malocclusion, where an end-on relationship exists between molars and canines (Figure 4).

The treatment protocol comprises two phases, incorporating regular oral prophylaxis. Addressing the Class II skeletal base malocclusion involves the utilization of Andreasen's modified activator, in conjunction with pre-adjusted fixed appliances adhering to McLaughlin, Bennett, and Trevisi's 0.022 slot prescription.

**Figure 5:**
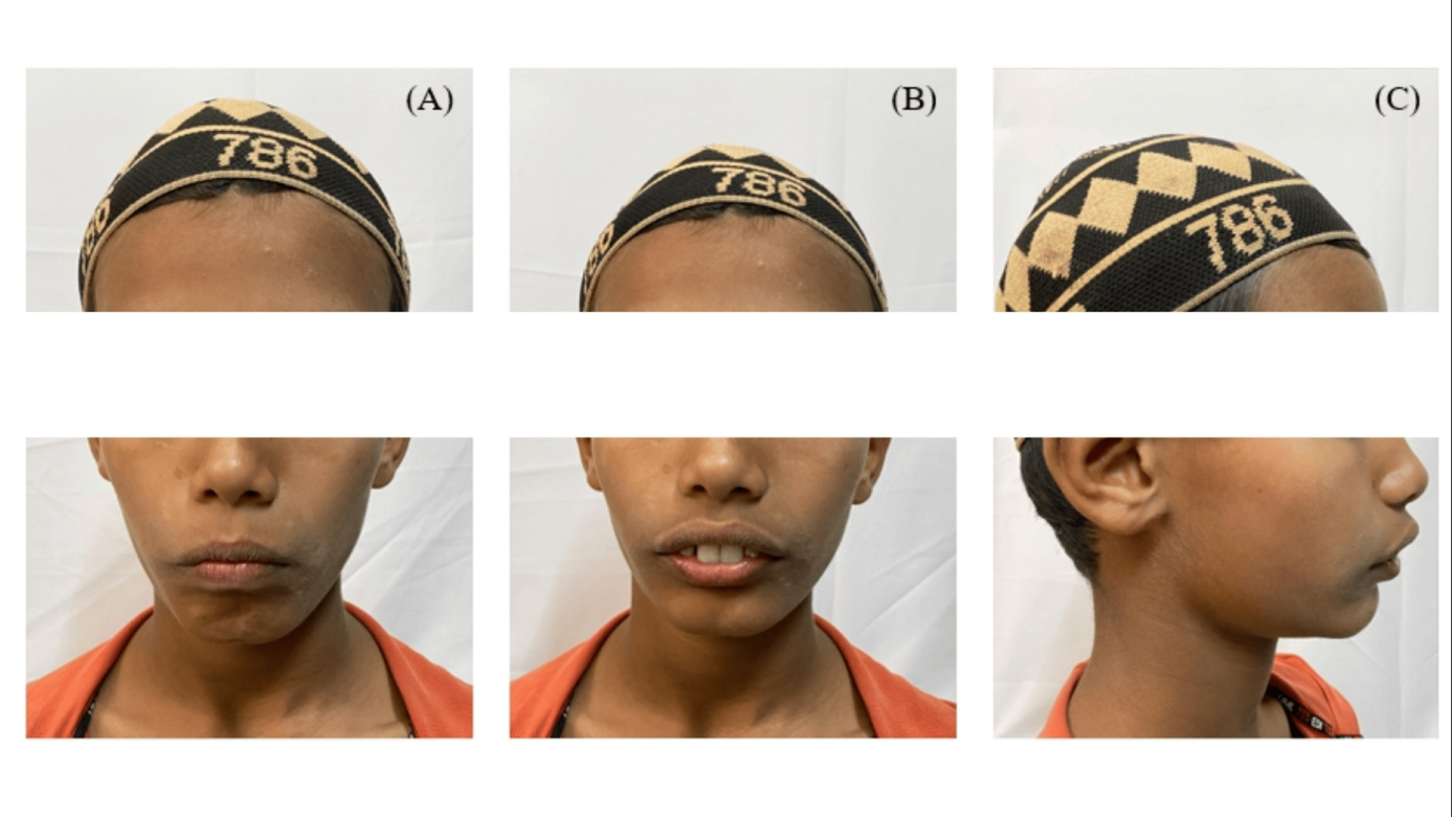
Extraoral photographs with an appliance showing the (A) frontal, (B) smiling, and (C) profile views

**Figure 6:**
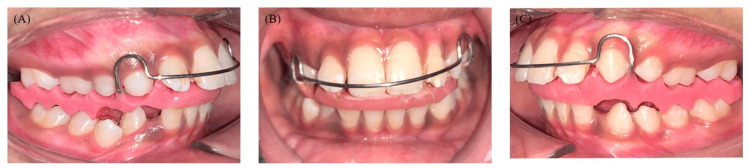
Intraoral photographs with an appliance showing the (A) right lateral, (B) frontal, and (C) right lateral views

The growth modification procedure, employing a myofunctional appliance, aims to rectify the skeletal Class II pattern, attain a Class I skeletal and dental relationship, establish appropriate overjet and overbite, diminish the convex profile, and enhance lip competence. A construction bite is an intermaxillary wax record used to relate the mandible to the maxilla to improve the skeletal inter-jaw relation. A horseshoe-shaped wax rim is made in the proper arch form, and it should be 2-3 mm thicker than the planned construction bite for guiding the mandible. Wax is placed in the lower arch to guide the mandible into the desired position without exerting force. An edge-to-edge incisal relation is maintained. The patient was prescribed a myofunctional appliance (activator) to facilitate these goals, and follow-up appointments should be scheduled every six weeks (Figures 5, 6) [7].

**Figure 7:**
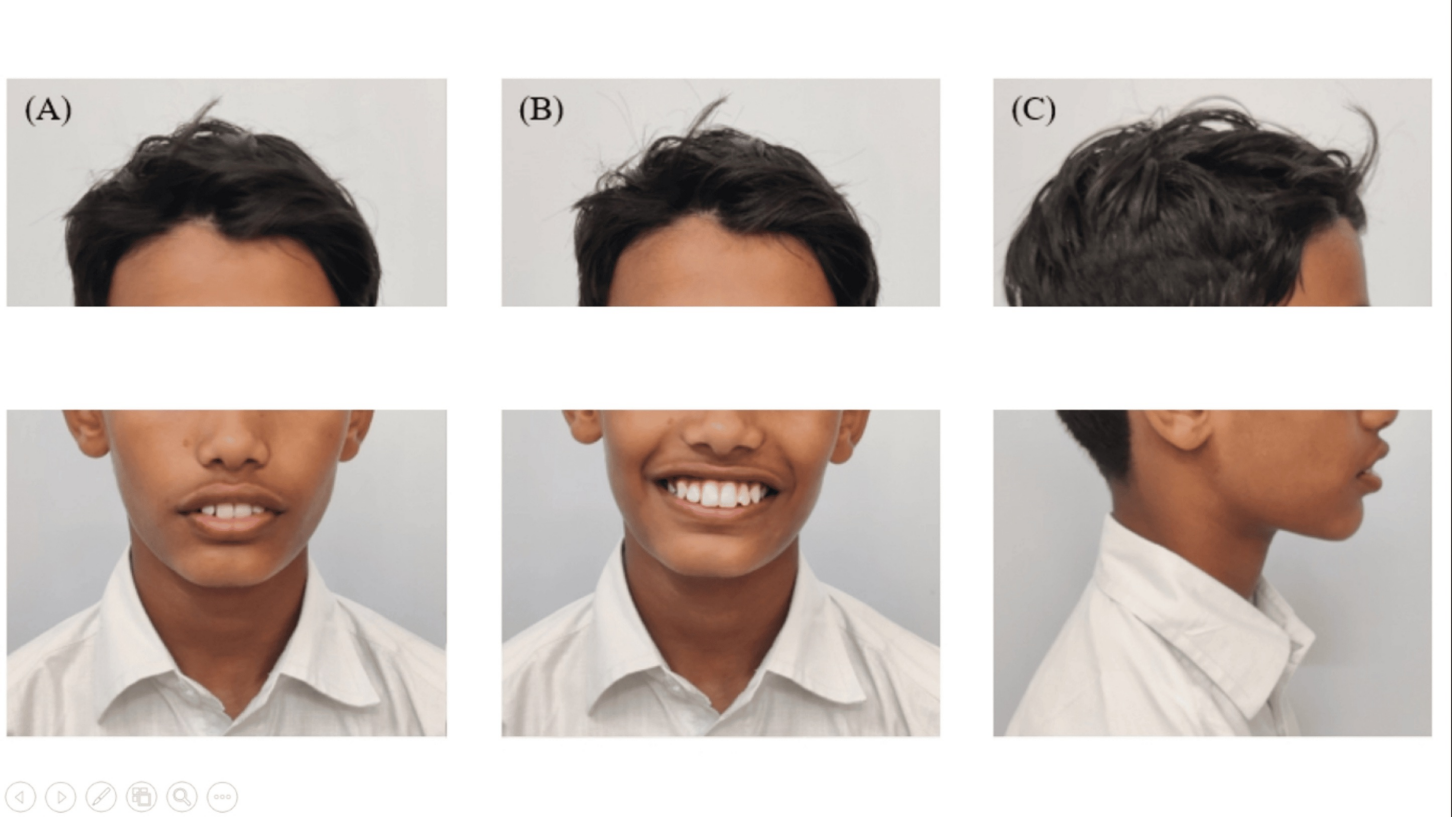
Posttreatment extraoral photographs showing the (A) frontal, (B) smiling, and (C) profile views

After undergoing myofunctional therapy (Figure 7), the patient's facial profile became more balanced, with a notable increase in the lower one-third facial height.

**Figure 8:**
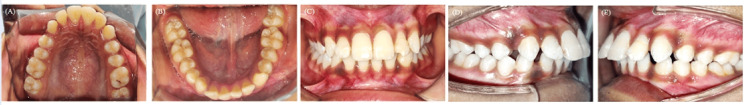
Posttreatment extraoral photographs showing the (A) maxillary arch, (B) mandibular arch, (C) anterior in occlusion, (D) right lateral occlusion, and (E) left lateral occlusion

The soft tissue profile improved, and there was a significant reduction in severe overjet and deep impinging overbite, resulting in Class I skeletal and dental relationships (Figure 8).

**Figure 9:**
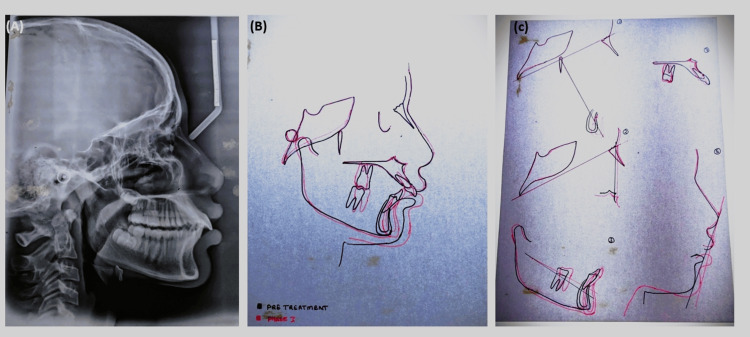
Postoperative lateral cephalogram evaluation showing the (A) postoperative lateral cephalogram, (B) superimposition of pretreatment and postoperative cephalogram, and (C) superimposition of planes with the help of cephalogram

A cephalometric analysis following myofunctional therapy revealed a favorable mandibular advancement, leading to a decrease in skeletal Class II (Figure 9).

## Discussion

These dentoalveolar and skeletal changes were attributed to various factors: inhibition of maxillary growth, correction of the lower lip biting habit, remodeling of the temporomandibular joint, and a natural increase in mandibular bone mass. These dentoalveolar changes contributed to achieving Angle's Class I relationship, aided by precise bite registration. Habit correction involved using a lower lip pad to keep the lower lip away, pacifying hyperactive mentalis muscle activity.

The activator appliance, used in functional appliances to advance the mandible sagittally while controlling the vertical dimension, enhances the activity of the protractor and elevator muscles while simultaneously relaxing and stretching retractors. This leads to a more favorable muscle pattern and induces changes in bony structures as the muscles adapt to new functional stresses.

Functional appliances such as the activator have several effects in treating skeletal Class II malocclusion, including reducing the ANB angle, restricting maxillary growth, advancing the mandible, increasing lower facial height, correcting overjet, improving overbite, uprighting maxillary incisors, protruding mandibular incisors, correcting dental Class II malocclusion, reducing facial convexity, and decreasing the mentolabial fold. The activator helps to eliminate abnormal perioral muscle function, which interferes with muscle growth and makes it easier to maintain oral hygiene. Patient cooperation while wearing an appliance is important [7].

According to McNamara and Carlson (1979), experimental animal studies have histologically demonstrated increased condylar growth resulting from mandibular hyperpropulsion [8]. Owen (1981) reported cephalometrically increased sagittal plane mandibular growth, which contributes to correcting Class II malocclusion by promoting greater forward mandibular growth than naturally expected increments [9].

According to the findings of Chabre's (1900) study, the activator appliance is reported to generate a force system resulting in negative moments, thereby inducing a clockwise rotation of the palatal and occlusal planes by passing behind the centers of resistance of both the maxilla and the upper alveolar process [10]. Moreover, as per the research by Harvold and Vargervik (1971), the activator appliance is associated with 1.4 mm of lingual tipping in maxillary incisors and 0.5 mm of labial tipping in mandibular incisors, potentially contributing to Class I malocclusion by hindering maxillary dentoalveolar growth and fostering mesial development of mandibular dentoalveolar structures [11].

## Conclusions

Myofunctional therapy employing an activator appliance has been recognized as an efficacious intervention for addressing Class II Division 1 malocclusion in children with mixed dentition and poor oral habits. The primary objective of this therapeutic modality is the correction of malocclusion and the enhancement of facial aesthetics. The findings of the study assert the plausibility of early intervention tailored to individual patient preferences and compliance. It is recommended to further scrutinize and incorporate this strategy into forthcoming treatment methodologies and research endeavors.
